# Deletion of Endo-β-1,4-Xylanase *VmXyl1* Impacts the Virulence of *Valsa mali* in Apple Tree

**DOI:** 10.3389/fpls.2018.00663

**Published:** 2018-05-17

**Authors:** Chunlei Yu, Ting Li, Xiangpeng Shi, Muhammad Saleem, Baohua Li, Wenxing Liang, Caixia Wang

**Affiliations:** ^1^Key Lab of Integrated Crop Pest Management of Shandong Province, Shandong Provincial Key Laboratory of Applied Mycology, College of Plant Health and Medicine, Qingdao Agricultural University, Qingdao, China; ^2^Department of Plant and Soil Sciences, University of Kentucky, Lexington, KY, United States

**Keywords:** *Valsa mali*, endo-β-1, 4-xylanase, gene characterization, gene deletion, virulence, apple tree

## Abstract

*Valsa mali*, a parasitic fungus, is a destructive pathogen of apple tree that causes heavy economic losses in China. The pathogen secretes various cell wall-degrading enzymes (CWDEs) that degrade plant cell-wall components, and thus facilitate its entry into host cells. Therefore, functional analysis of the genes encoding CWDEs is necessary to understand virulence of *V. mali* toward apple tree. Here, we identified and cloned an endo-β-1,4-xylanase gene, *VmXyl1* in *V. mali*. The full-length cDNA of *VmXyl1* is 1626 bp containing 5′- and 3′-non-coding regions, as well an open reading frame of 1320 bp that encodes a protein with a calculated molecular mass and an isoelectric point of 43.8 kDa and 4.4, respectively. The predicted amino acid sequences showed significant homology to a family GH10 of glycosyl hydrolases. The apple branch extract and beechwood xylan, but not glucose, induced the expression of *VmXyl1*. Furthermore, *VmXyl1* had high expression levels in the apple tree bark during the pathogen infection. The deletion of *VmXyl1* did not affect mycelia growth; however, it significantly reduced pycnidia formation in *V. mali*. The deletion strains showed a reduced virulence toward apple leaves and twigs. Moreover, the mutant strains had reduced endo-β-1,4-xylanase activity and growth when cultured using beechwood xylan as the only carbon source. Reintroducing wild-type *VmXyl1* into the mutant strains rescued the defect phenotype. We conclude that *VmXyl1* determines the virulence of *V. mali* toward apple tree. These results provide valuable insight into the plant–pathogen molecular interactions.

## Introduction

Phytopathogens negatively impact plant performance (Wang et al., 2012; Saleem et al., 2017), and thus limit global tree fruit production (Reganold et al., 2001). The apple tree canker fungus, *Valsa mali* (anamorph *Cytospora* sp.), represents a significant threat to the apple production in eastern Asia, especially in China (Cao et al., 2009; Wang et al., 2013; Li et al., 2015). It causes elongated cankers on tree branches and trunks that ultimately lead to the death of the whole plant (Wang et al., 2012). Mostly, the infected bark tissues develop two types of symptoms: (i) reddish-brown, alcohol-smelling, and ulcer type softened lesions; and (ii) branch or twig dieback. During the late stage of disease, *V. mali* produces pycnidia on the cankers that release conidia throughout the year (Li et al., 2013). Given that pathogen infection expands rapidly through the xylem, fungicide use often fails to control apple tree valsa canker (Abe et al., 2007). A limited understanding of the virulence mechanism of *V. mali* limits our ability to control the disease. Therefore, it is urgently important to understand the virulence mechanism of *V. mali*. It may provide a foundation for developing more effective disease-management strategies.

Similar to other phytopathogenic fungi, *V. mali* secretes several toxic compounds and cell-wall-degrading enzymes (CWDEs) throughout the infection process to degrade defensive barriers and kill the plant cells (Chen et al., 2012; Wang et al., 2014; Yin et al., 2015). The CWDEs break down plant cell wall, and thus provide assimilable nutrients to facilitate pathogen entry and disease development (Nguyen et al., 2011; Morales-Cruz et al., 2015; Pérez-Hernández et al., 2017). A whole-genome analysis also revealed that *V. mali* contains a number of genes associated with plant cell wall-degradation and secondary metabolite biosynthesis (Yin et al., 2015). Some studies investigated the role of CWDEs genes and enzymes such as pectate lyases and polygalacturonases in the virulence of *V. mali* (Xu et al., 2016; Xu C.J. et al., 2017; Xu M. et al., 2017). Despite being one third of the plant cell wall of hemicellulose (Collins et al., 2005), the role of hemicellulose-degrading enzymes in *V. mali* virulence, remains understudied.

The xylan, a carbohydrate, is composed of β-1,4-D-xylose residues. It is a major hemicellulosic component of the plant cell wall (Collins et al., 2005). The endo-β-1,4-xylanases (EC 3.2.1.8) play a crucial role in the hydrolysis of xylan by cleaving β-1,4 linkages of the xylosyl backbone (Nguyen et al., 2011). These xylanases belong to either family F (GH10) or family G (GH11). The family F contains high-molecular-mass xylanases whereas family G contains lower molecular mass xylanases (Biely et al., 1997). The xylanase-encoding genes are studied in some fungal pathogens to determine their role in cell wall-degradation, though most of these have nothing to do with the virulence (Apel et al., 1993; Gómez-Gómez et al., 2001; Wu et al., 2006; Sella et al., 2013). However, endo-β-1, 4-xylanase-encoding genes *xyn11A* and *SsXyl1* are prerequisite for virulence of *Botrytis cinerea* and *Sclerotinia sclerotiorum* on host plants, respectively (Brito et al., 2006; Yu et al., 2016). Most of the identified genes encoding xylanases in fungal pathogens belong to the family GH11. However, the role of xylanase-encoding genes belonging to family GH10 in the fungal virulence remains understudied.

The endoxylanases play a significant role in plant cell wall degradation whereas their activities are often correlated to the virulence and pathogenicity of *V. mali* strains (Chen et al., 2012; Li et al., 2014). Here, we report the cloning of a full-length cDNA gene, *VmXyl1*, which encodes an endo-β-1,4-xylanase of *V. mali*. We disrupted this gene in *V. mali* and then studied the phenotypic and epidemiological characteristics of the mutant strains. In particular, we demonstrated the role of *VmXyl1* in fungal pathogen invasion, expansion and disease development in apple tissues. Our results may enhance understanding of the *V. mali* virulence toward apple tree, and thus may help to develop disease control strategies.

## Materials and Methods

### Fungal Strains and Culture Conditions

We isolated *V. mali* wild-type strain LXS080601 from an infected Fuji (*Malus domestica* Borkh. cv. ‘Fuji’) apple tree in Qixia, Shandong Province. The strain was maintained on potato dextrose agar (PDA, 200 g of potato, 20 g of dextrose, and 15 g of agar per liter) at 25°C for routine use. The conidial suspensions were prepared from *V. mali* cultures on barley medium (70 g of barley, 20 ml of 6% honey solution, and 20 ml of 1% peptone) (see details, Zhao et al., 2012). The gene deletion transformants and complemented strains were cultured on PDA supplemented with 100 μg/ml hygromycin B or G-418 (Sigma, St. Louis, MO, United States). The experimental medium contained 3.0 g of NaNO_3_, 1.0 g of KH_2_PO_4_, 0.5 g of KCl and MgSO_4_⋅7H_2_O, and 0.01 g of FeSO_4_ per liter at a pH of 5.8. Further, we added different sole carbon sources such as glucose (2%), beechwood (2%), oat spelt xylan (Sigma, St. Louis, MO, United States), and apple branch extract (20%) into the synthetic medium (Wang et al., 2014). We inoculated synthetic medium containing different sole carbon sources with conidial suspensions to induce CWDEs (Wang et al., 2014). The measured colony radius was used to calculate the growth rate of different strains on solid medium (1.5% agar). Unless otherwise stated, we used either PDA or synthetic media containing 2% beechwood xylan or 20% apple branch extract as sole carbon sources.

### Cloning of *VmXyl1* in *V. mali*

The genomic DNA was extracted from *V. mali* LXS080601 mycelium as described by Zhang et al. (2007). The total RNA was extracted from LXS080601 using the RNAiso Plus Kit (TaKaRa, Dalian, China) according to the manufacturer’s protocol. The cDNA was synthesized using the Prime Script^TM^ RT reagent Kit with gDNA Eraser (TaKaRa, Dalian, China) with an oligo (dT)_12-18_ primer. One gene, namely *VmXyl1*, with putative xylanase activity and high expression level during *V. mali* infection was cloned. The primers used in this study were synthesized by Sangon (Shanghai, China) (Supplementary Table S1). The 3′-Full RACE Core Set (TaKaRa, Dalian, China) and SMARTer^TM^ 5′ RACE cDNA Amplification Kit (Clontech, Mountain View, CA, United States), were used to clone 3′-end and 5′-end cDNA fragments, respectively. We used the primer pair *VmXyl1*F/*VmXyl1*R to amplify open reading frame (ORF) of *VmXyl1*.

### Sequence Analysis and Phylogenetic Analysis

We performed sequence alignments of *VmXyl1*, and other reported xylanases gene of fungi using DNAMAN (version 6.0) with all the parameters set at the default values. Conserved amino acids were shown with a shaded background. A phylogenetic tree was constructed using the distance-based Neighbor-Joining method with MEGA (version 5.1). The signal peptide sequence and conserved domain were predicted using the Signal P4.1 Server and PFAM, respectively (Petersen et al., 2011).

### Detection of Gene Expression

To compare the expression level of *VmXyl1* in different carbon sources, a suspension containing 10^6^ conidia of *V. mali* wild-type strain was germinated at 25°C for 24 h in 100 ml of synthetic medium containing glucose, xylan, or apple branch extract. For *VmXyl1* and other seven endoxylanase genes from family GH10 and GH11 expression *in planta*, 1-year-old apple twigs were wounded as described by Xu M. et al. (2017). Mycelium plugs (*d* = 5 mm) from actively growing colony margins of the wild-type, gene deletion, and complemented strains were inoculated into the wounds. For samples at 0 hpi, bark tissues around inoculation sites containing mycelium plugs were collected. The junction of the healthy and infected apple bark tissues was sampled at different time points (6, 12, 24, 48, 72, and 168 h). The RNAiso Plus Kit (TaKaRa, Dalian, China) was used to extract RNA from the frozen plant tissues and mycelia in liquid nitrogen, and then the first-strand cDNA was synthesized. We used RT-PCR to determine the expression of *VmXyl1* in deletion and complementation strains with the gene-specific primer pair *VmXyl1*F/*VmXyl1*R to amplify a 1320-bp fragment (Supplementary Table S1). The PCR conditions were as following: 30 cycles of 94°C for 30 s, 55°C for 45 s, and 72°C for 60 s, with a final extension at 72°C for 5 min.

We determined the expression of *VmXyl1* and seven other endoxylanase genes in *planta* by qRT-PCR using gene-specific primers (Supplementary Table S1). All of the qRT-PCR experiments were conducted in a LightCycler^®^ 480II PCR Detection System (Roche, Germany) with SYBR Master Mix (TaKaRa, Dalian, China) following the manufacturer’s protocol. In order to normalize the expression levels, the *V. mali EF1-*α was used as an internal reference (Yin et al., 2013). The PCR cycle conditions consisted of an initial step of 30 s at 95°C followed by 40 cycles of 5 s at 95°C and 20 s at 60°C. We analyzed both threshold cycle (*C*t) and melting curves for each gene while the relative amounts of mRNA were calculated using 2^-ΔΔ^*^C^*^t^ method (Livak and Schmittgen, 2001). To confirm reproducibility of results, we used three biological and three technical replicates for each sample. We repeated the whole experiment thrice.

### Xylanase Activity Assays

To assay the xylanase activity of VmXyl1, the cDNA fragment encoding the amino acid of VmXyl1 (without signal peptide) was amplified and inserted into the pET 32a with a C-terminal 6 × His tag. The resulting vector was transformed into *Escherichia coli* strain Rosetta while the soluble recombinant protein was obtained after induction with 0.5 mM isopropyl s-thiogalactopyranoside (IPTG) for 16 h at 15°C (Shi et al., 2015). We used Ni-NTA Spin Column (Qiagen, Beijing, China) to purify the recombinant protein containing a polyhistidine (6 × His) sequence following the manufacturer’s instruction. To determine xylanase activity of the wild-type, gene deletion and complemented strains from the various carbon source treatments, we collected culture filtrate at the day 3 of inoculation. For xylanase activity during fungal infection, apple twigs were inoculated with the strains and samples were harvested at different time points (0, 1, 3, 7, 11, and 14 days) (Chen et al., 2012).

The xylanase activity was measured by the 3,5-dinitrosali cylicacid (DNS) method as described by Yu et al. (2016) with some modifications. The reaction mixture, consisting of 500 μl of samples (purified recombinant protein or culture filtrate), 2.5 ml 0.5% beechwood xylan in 50 mM sodium citrate buffer (pH 5.0), was incubated for 30 min at 50°C. Then, we added 3 ml DNS solution to the reaction mixture followed by boiling for 5 min. We measured absorbance at 540 nm. One unit of xylanase activity was equal to the amount of enzyme catalyzing the formation of 1.0 mmol of xylose per minute at pH 5.0 and 50°C. The activity was expressed as units per min per ml (U/ml) or units per min per gram of fresh weight (U/g⋅FW).

### Generation of Gene Deletion and Complementation Strains

The strategy used for constructing the gene deletion cassette was derived from the double-joint PCR method with some modifications (Yu et al., 2004). To generate the *VmXyl1* gene deletion mutants, we replaced *VmXyl1* with hygromycin phosphotransferase (*HPH*) (Supplementary Figure S2A). Upstream and downstream fragment of the *V. mali VmXyl1* gene was amplified from genomic DNA of wild-type LXS080601 using two sets of gene-specific primer pairs, *VmXyl1*PF/*VmXyl1*PR and *VmXyl1*TF/*VmXyl1*TR (Supplementary Table S1). Special *VmXyl1*PR and *VmXyl1*TF chimeric primers for gene contained the homologous joints to *HPH*. The HPH fragment was amplified from the plasmid pGI-3C using the primers *HPH*F/*HPH*R. The upstream, *HPH*, and downstream fragments were fused at a ratio of 1:3:1. Then, the gene deletion cassette was directly amplified using primers *VmXyl1*PF/*VmXyl1*TR. The PCR conditions consisted of an initial step of 60 s at 95°C followed by 15 cycles of 20 s at 95°C and 5 min at 58°C. The gene knock-out cassette was confirmed by sequencing.

The protoplast preparation and PEG-mediated transformation were conducted as described by Rollins (2003). We mixed regenerated mycelia with 10 ml of molten bottom agar containing 50 μg/ml hygromycin B. After 10 h cultivation at 25°C in dark, we overlaid top agar containing 100 μg/ml hygromycin B. After 3–5 days, transformants were picked and inoculated onto the PDA containing 100 μg/ml hygromycin B. The PCR detection of *VmXyl1* deletion mutants was carried out by amplification with gene-specific primer pairs to verify the knock-out of *V. mali VmXyl1* and insertion of *HPH*. We used two independent knock-out lines (Δ*VmXyl1* and Δ*VmXyl1.2*) in all experiments. Since the phenotypes of both independent mutants were identical, only the results of Δ*VmXyl1* are shown in most figures for clarity and simplicity.

To construct the gene complement vector, we inserted a neomycin-resistance gene into pCAMBIA3301 at the *Xba*I site to produce p3300NEO. A 3.2 kb fragment was amplified from the genome DNA of LXS080601 strain with the primer pair *VmXyl1*CF/*VmXyl1*CR carrying *Eco*RI and *Xho*I digested sites, respectively (Supplementary Table S1). The fragment constrained the entire *VmXyl1* coding sequence and 912 bp of 3′ and 953 bp of 5′ untranslated region (UTR) (Supplementary Figure S2A). The fragment was digested with *Eco*RI and *Xho*I and cloned into p3300NEO to produce the p3300NEO *VmXyl1*-Com. The vector was then linearized with *Xho*I and transformed into the Δ*VmXyl1* protoplasts with PEG-mediated transformation.

### Pathogenicity Assays

We performed pathogenicity assays on the leaves of a 1-year-old apple twig (*M. domestica* Borkh. cv. ‘Fuji’) taken from our greenhouse at Qingdao Agricultural University, Qingdao, China. Then, we sterilized the detached leaves and twigs with 75% ethanol, and evenly distributed wounds were made as described by Wang et al. (2013, 2014). Mycelium plugs (*d* = 5 mm) from actively growing colony margins of the wild-type, gene deletion, and complemented strains were used to inoculate the wounds. The inoculated leaves and twigs were placed in trays to maintain humidity at 25°C in the dark. We measured lesion length and performed photography at different time intervals. These assays were repeated thrice, with at least 10 leaves and twigs per treatment.

### Statistical Analysis

All statistical analysis was conducted using SPSS software (Version 17.0, SPSS Inc., Shanghai, China). All data were subjected to analysis of variance (ANOVA) followed by Duncan’s multiple range tests. The asterisks indicate a statistically significant difference with the control (wild-type strain) (*p* = 0.05).

## Results

### Cloning and Sequencing of *VmXyl1*

**FIGURE 1:**
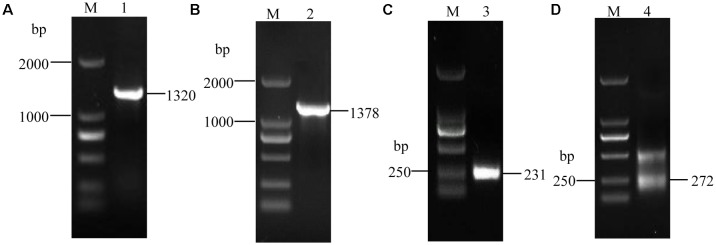
The electrophoretogram of *VmXyl1* cloning process from the *Valsa mali*. **(A)** The RT-PCR amplification of the open reading frame of *VmXyl1*. The lane M represents DNA marker DL2000. In lane 1, a 1320 bp fragment was obtained using the primer pair *VmXyl1*F/*VmXyl1*R with the first-strand cDNA as a template. **(B)** The PCR amplification of DNA fragment of the *VmXyl1*. In lane 2, a 1378 bp fragment was obtained with genomic DNA as the template. **(C)** The cloning of *VmXyl1* by the 3′-RACE. In lane 3, a 231 bp fragment was obtained with the primer pair Inner Primer/IN3RC. **(D)** The cloning of *VmXyl1* by the 5′-RACE with the primer pair NGSP1/UPM. In lane 4, two fragments were obtained in the nested PCR and a 272 bp band was the 5′-end cDNA region of *VmXyl1*.

Using the synthesized cDNA and genomic DNA as the templates, we amplified one fragment of approximately 1.3 kb using the ORF primers, *VmXyl1*F/*VmXyl1*R (**Figures 1A,B**). The cDNA and genomic clones of *VmXyl1* were agarose-gel-purified and then cloned into the pMD18-T vector. The sequence analysis verified that the cDNA fragment of ORF was 1320 bp in length whereas the DNA fragment was of 1378 bp in length, with only one short intron of 58 bp. Using the 3′-RACE kit, we amplified the first-round PCR with a primer pair (Out Primer/OT3RC). After re-amplifying first-round PCR product with a primer pair (Inner Primer/IN3RC), we obtained one PCR product in the second round PCR (**Figure 1C**). The sequence analysis of this product confirmed that the cloned 3′-end cDNA fragment is exactly 231 bp in length, with a 131 bp 3′-untranslated region (3′-UTR, except for the polyA tail). As shown in **Figure 1D**, we amplified two fragments in the second-round PCR using the 5′-RACE kit with primer pairs GSP1/NUP and NGSP1/UPM for the first- and second-round PCR, respectively. The sequence analysis confirmed that the length of the 5′-end cDNA is 272 bp, with a 175 bp 5′-untranslated region (5′-UTR).

### VmXyl1 Characterization and Phylogenetic Analysis

**FIGURE 2:**
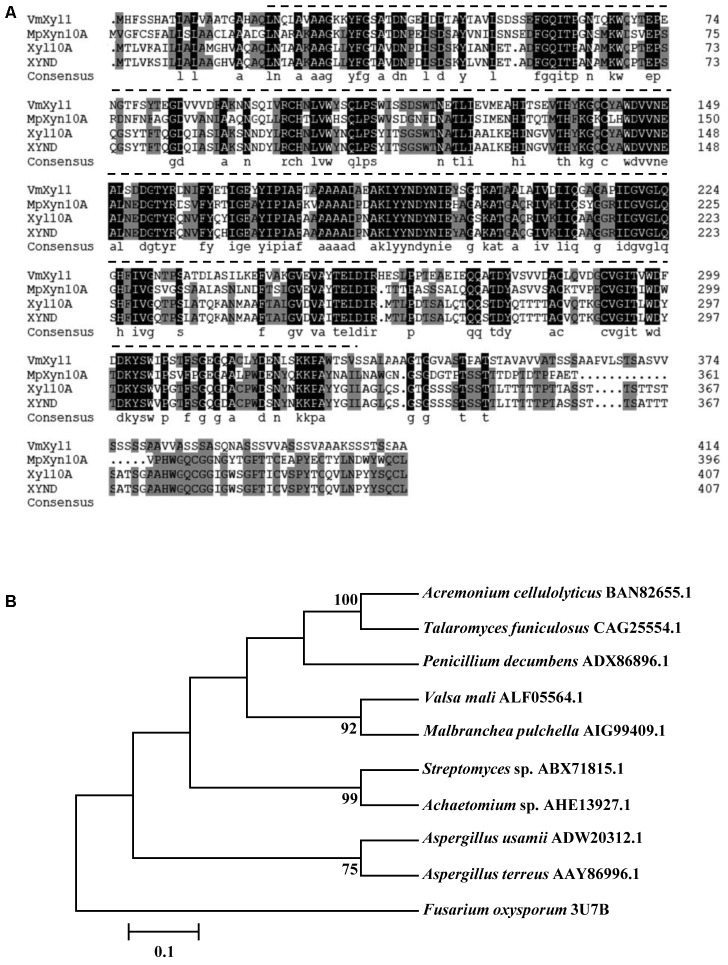
Multiple alignments and phylogenetic tree of the amino acid sequences of VmXyl1 in comparison to the sequences of other endoxylanases from family GH10. **(A)** Alignments of the VmXyl1 with three well-characterized endoxylanase proteins of the family GH10. Identical or similar residues are shown with black or gray background, respectively. Protein accession numbers are as follows: AIG99409.1 (*Malbranchea pulchella*, MpXyn10A), CAG25554.1 (*Talaromyces funiculosus*, XYND) and BAN82655.1 (*Acremonium cellulolyticus*, Xyl10A). **(B)** Phylogenetic analysis of VmXyl1 with nine well-characterized endoxylanase proteins of the family GH10. The sequences were analyzed using the ClustalW2 tool and the tree was generated by the Neighbor-Joining method using MEGA 5.1 software. Confidence levels above the nodes were obtained from a 1000 bootstrap analysis program. Species names are followed by accession numbers of endoxylanase genes.

The *VmXyl1* gene contains an ORF of 1320 bp, encoding a 439-amino acid protein. Using the Signal P4.1 server, the N-terminal of *VmXyl1* was predicted to contain a typical signal peptide (Petersen et al., 2011). The Signal P4.1 predicted an unambiguous signal peptide cleavage site between amino acid Gln^20^ and Leu^21^, thus indicating that VmXyl1 is a secretory protein. The putative mature protein has a molecular mass of 43.80 kDa and an isoelectric point (pI) of 4.42, in which four *N*-glycosylation sites were present. However, the *O*-glycosylation site was not found. The residues 21–332 in the protein were predicted by Pfam (Finn et al., 2015) to be a Glyco_hydro_10 glycosyl hydrolase motif (**Figure 2A**).

The sequence comparison using Blastp in GenBank showed that VmXyl1 exhibited high similarities with well-characterized endo-β-1,4-xylanase proteins belonging to the GH10 family of glycosyl hydrolases. The multiple alignments analysis of VmXyl1 (GenBank ALF05564.1) revealed that VmXyl1 shared 53% sequence identity with MpXyn10A from *Malbranchea pulchella*, which is a thermostable xylanase GH10 (Ribeiro et al., 2014). The sequence alignment and phylogenetic tree are shown (**Figure 2**).

To confirm the xylanase activity, we constructed the *VmXyl1* cDNA (without signal peptide) into a pET32a expression vector. The resulting vector was transformed into *E. coli* strain Rosetta (Shi et al., 2015). We got the purified recombinant protein by using Ni-NTA Spin Column, and then the xylanase activity was determined. The enzyme activity of the recombinant protein was 2.63 U/ml. This result indicated that *VmXyl1* encodes a xylanase in *V. mali*.

### *VmXyl1* Expression Pattern

**FIGURE 3:**
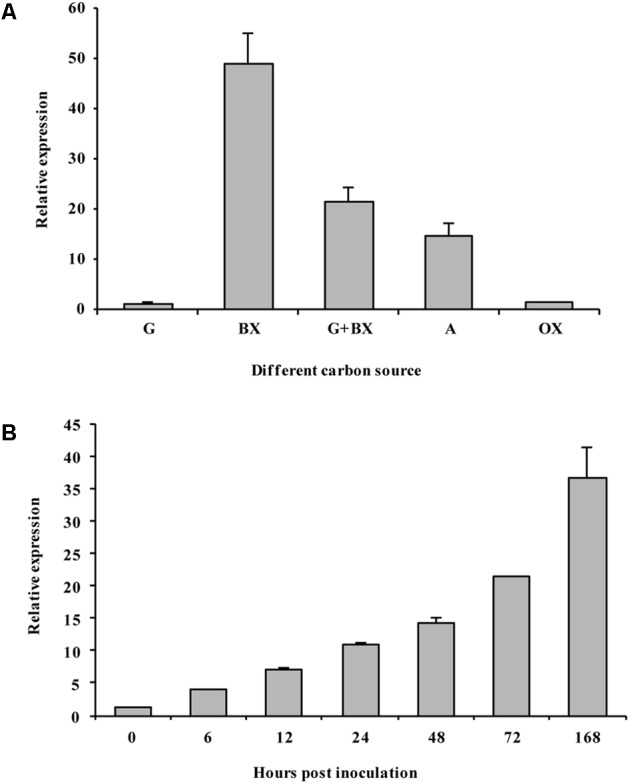
The expression of *VmXyl1* in wild-type stain LXS080601 under different growth conditions. **(A)** The levels of *VmXyl1* mRNA in each sample were normalized to the levels of *EF1-α* mRNA. The bars (G, BX, G+X, A, and OX) represent the expression of *VmXyl1* mRNA in the wild-type strain after 24 h cultivation in the synthetic medium with glucose, beechwood xylan, glucose + beechwood xylan, apple branch extract, and oat spelt xylan as sole carbon sources, respectively. The relative level of *VmXyl1* mRNA in the mycelia cultured in medium containing glucose as carbon source was normalized to one. **(B)** Levels of *VmXyl1* mRNA in the apple bark infected with *V. mali* strain LXS080601 at various hours post inoculation (hpi). Mycelia grown on PDA for 3 days were used to inoculate, and then the junction of the healthy and infected apple bark was collected. For samples at 0 hpi, bark tissues around inoculation sites containing mycelium plugs were collected. The relative abundance of *VmXyl1* mRNA in the mycelia grown on PDA for 3 days was normalized to one. The means and standard error of the expression levels were calculated from three independent biological replicates. Bars represent the standard error.

We determined the expression of *VmXyl1* in the culture medium using qRT-PCR. The mRNA transcript levels of *VmXyl1* in wild-type strain grown in beechwood xylan and apple branch extract were significantly higher than in the media containing glucose or oat spelt xylan (**Figure 3A**). We observed the maximal level of transcript in cells grown in the beechwood xylan-containing medium. Overall, both beechwood xylan and apple branch extract induced the expression of *VmXyl1*. We also detected the xylanase activity in the culture medium supplemented with different carbon sources (Supplementary Figure S1). The pattern of *VmXyl1* expression was same as that of the enzyme activity. The enzyme activity was almost undetectable in the glucose- or oat spelt xylan-supplemented media. The enzyme activity was maximal in the medium containing 2% beechwood xylan whereas it declined with the decreasing xylan concentration (Supplementary Figure S1).

We also determined the levels of *VmXyl1* mRNA during the infection process of apple bark with *V. mali* (**Figure 3B**). To do this, we sampled infected apple bark tissue at different hours post inoculation (hpi), and then compared transcript levels to those of mycelia grown on PDA for 3 days. In the early phase of infection (6 hpi), we observed a slight induction in the *VmXyl1* mRNA levels (nearly fourfold increase). The *VmXyl1* expression levels gradually increased during the infection (6–72 hpi) while it increased dramatically afterwards (36.7-fold increase, at 168 hpi). Thus, the high induction of *VmXyl1* during infection indicates a potential role of in the pathogenicity of *V. mali*, especially at the stage of lesion expansion.

### The Effect of *VmXyl1* on Vegetative Growth and Pycnidia Formation

For functional analysis of the *VmXyl1* in *V. mali*, we obtained the knock-out cassette of *VmXyl1* by double-joint PCR and transformed the protoplasts of the wild-type strain by the PEG-mediated method. We selected several transformants that grew stably on hygromycin-containing medium and were further tested by the genomic PCR (Supplementary Figure S2). Two strains were selected randomly, and the *VmXyl1* expressions were determined using RT-PCR. The recombinant strains Δ*VmXyl1*, and Δ*VmXyl1.2* lacked *VmXyl1* transcript (Supplementary Figure S2). The complementation of *VmXyl1* deletion mutants was performed by generating a construct that was transformed into the protoplasts of Δ*VmXyl1* and Δ*VmXyl1.2.* The complementation was confirmed using genomic PCR and RT-PCR (Supplementary Figure S2).

**FIGURE 4:**
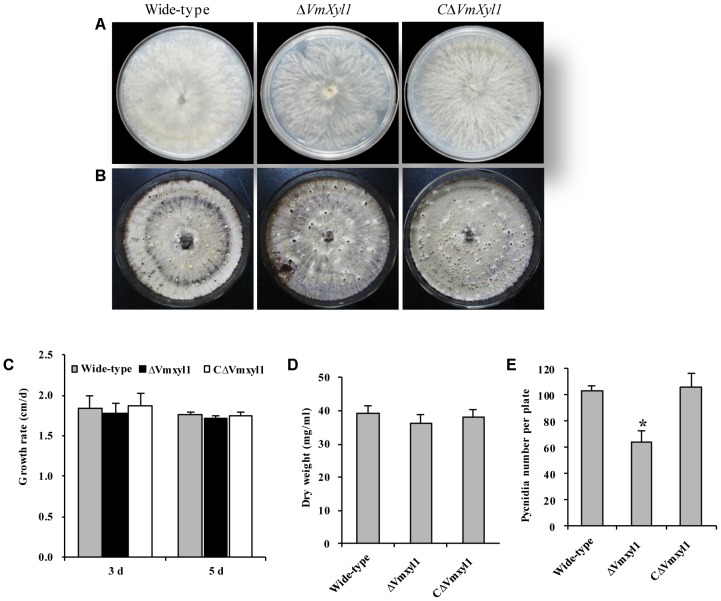
The growth and development of wild-type and mutants. **(A)** The colony phenotype of the wild-type, Δ*VmXyl1* and *C*Δ*VmXyl1* strains growing on potato dextrose agar (PDA) medium at 25°C for 5 days. **(B)** Pycnidia formation of strains on PDA medium induced by the UV-light (365 nm) at 25°C for 30 days. **(C)** The growth rate of strains on PDA medium. **(D)** The mycelial dry weight of strains in potato dextrose broth (PDB) medium for 7 days with 150 rpm/min at 25°C. **(E)** Number of pycnidia produced in 9 cm per petri plates. Bars indicate standard deviation of means of three technical replicates. Asterisk on bars indicate a significant difference with the wild-type strain (*P* < 0.05).

To determine the role of *VmXyl1* in *V. mali* growth and development, we cultured the wild-type LXS080601 and the two mutant strains (Δ*VmXyl1*, *C*Δ*VmXyl1*) on PDA and potato dextrose broth (PDB) media. All strains exhibited similar colony morphology. Although having loose mycelia and a reduced apical extension rate, the mycelial growth rate of Δ*VmXyl1* strain did not differ statistically from other two strains on PDA (**Figures 4A–C**). Furthermore, the mycelium dry weight also did not differ statistically between the mutant strains after 7-day culturing in PDB medium (**Figure 4D**). All strains formed pycnidia; however, the strain Δ*VmXyl1* produced fewer pycnidia than the wild-type on PDA plates under UV-light (365 nm) at 25°C. The complementation of gene deletion mutant with *VmXyl1*, its promoter, and terminator restored the wild-type pycnidia formation (**Figures 4B,E**).

### *VmXyl1* Is Required for Virulence

**FIGURE 5:**
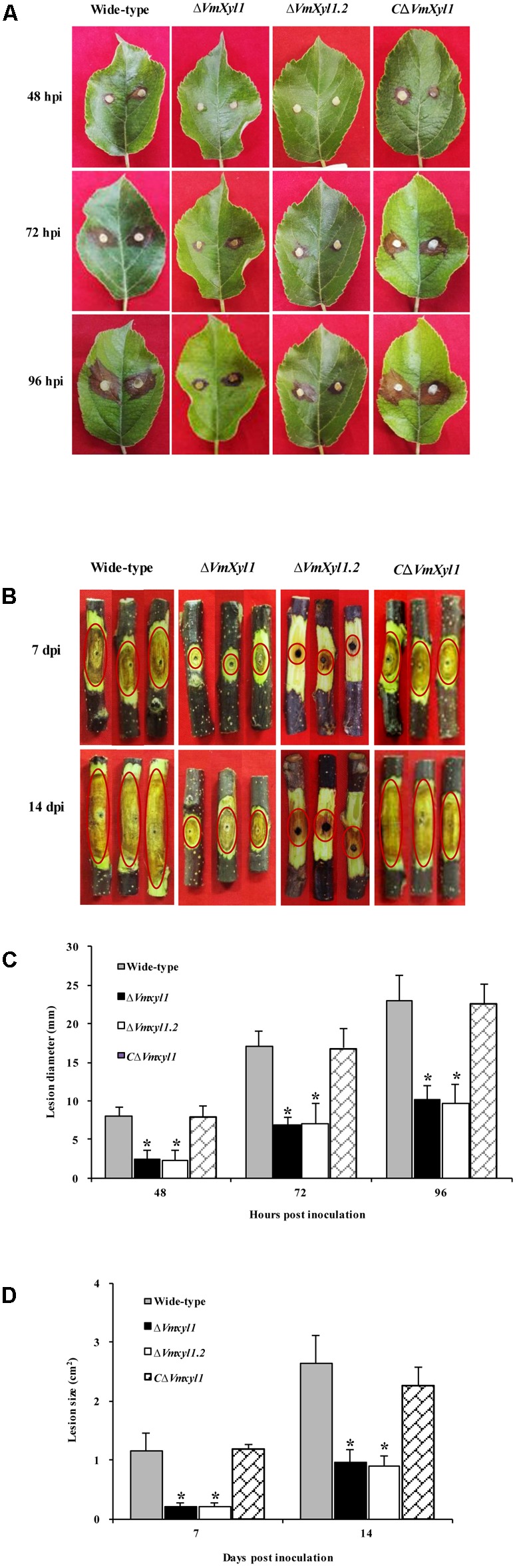
Comparison of the pathogenicity of wild-type LXS080601, *Vmxyl1* deletion and complementation mutants. **(A**,**B)** The infected phenotype of apple leaves and twigs inoculated with different strains at the indicated times. The red circles highlight the infection symptoms in apple twigs. **(C**,**D)** Diameters and area of lesions produced by different strains on apple leaves and twigs at different times. Asterisks on bars indicate a significant difference with the wild-type strain (*P* < 0.05). The experiments were repeated thrice.

The increased transcript levels of *VmXyl1* during pathogen infection and the endo-β-1,4-xylanase activity of VmXyl1 protein promoted us to investigate whether *VmXyl1* was involved in *V. mali* virulence. We performed pathogenicity assays of the wild-type, Δ*VmXyl1* and *C*Δ*VmXyl1* mutant strains on detached apple leaves and twigs (cv. ‘Fuji’). Then, we measured the lesions caused by these strains at different times after inoculation. The wild-type lesions expanded rapidly whereas the Δ*VmXyl1* mutant showed a reduced ability to infect and expand at 24 hpi on leaves and 3 days post inoculation (dpi) on the twig (data not shown). Slight lesions were found in the mutant-inoculated apple leaves at 48 hpi and twigs at 7 dpi (**Figures 5A,B**). The sizes of lesions were 8.1 mm and 1.2 cm^2^ in the wild-type inoculated leaves and twigs, respectively (**Figures 5C,D**). The Δ*VmXyl1* mutants demonstrated a more than 60% reduction in the average lesion size on apple leaves and twigs during infection (**Figures 5C,D**). The complementation strain *C*Δ*VmXyl1* of the deletion strain Δ*VmXyl1*, restored its virulence to the wild-type level. Overall, our results demonstrated that the virulence of the *VmXyl1* mutant was significantly impaired.

### Ability of *VmXyl1* Mutants to Use Xylan

**FIGURE 6:**
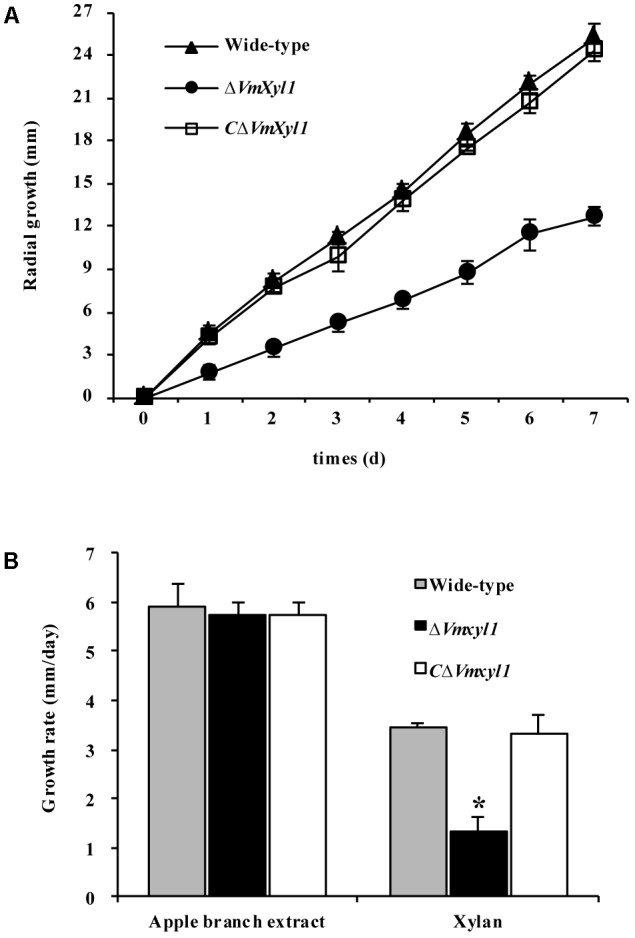
The growth of *VmXyl1* mutants in the medium containing beechwood xylan as a sole carbon source. **(A)** The radial growth of wild-type, Δ*VmXyl1* and *C*Δ*VmXyl1* strains in the petri dishes containing beechwood xylan was determined by measuring the colony diameters daily. **(B)** The growth rate was calculated for each strain on minimal medium containing either apple branch extract or beechwood xylan as sole carbon sources. Values plotted represent the mean and standard deviation of three technical replicates. Asterisks on bars indicate a significant difference with the wild-type strain (*P* < 0.05).

To test whether the deletion of *VmXyl1* determines the ability of *V. mali* to utilize xylan, we compared the growth of the wild-type and mutant strains on minimal solid medium containing 2% beechwood xylan as a sole carbon source. The deletion of *VmXyl1* significantly affected the growth rate of the mutant in the solid media; the deletion mutant grew ∼50% slower than the wild-type (**Figure 6A**). The rescued strain *C*Δ*VmXyl1*, however, grew at about the same rate as the wild-type, thus confirming that VmXyl1 protein was responsible for the phenotype. The growth rate on rich media, such as apple branch extract, was not significantly different among the strains (**Figure 6B**).

### Deletion of *VmXyl1* Affects Xylanase Activity

**FIGURE 7:**
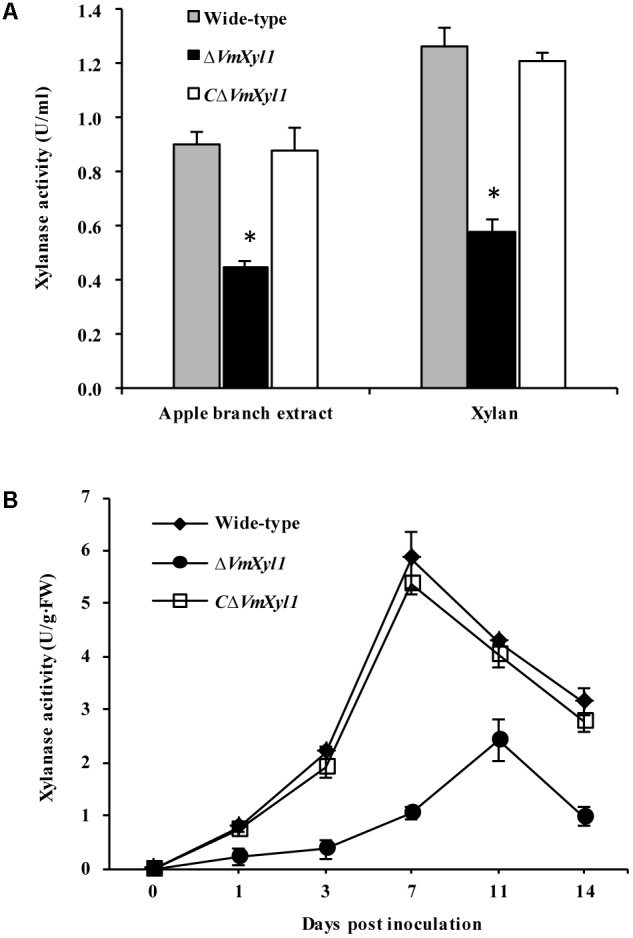
Effect of *VmXyl1* deletion on the β-1,4-xylanase activity. **(A)** Xylanase activity was determined in the medium containing apple branch extract or beechwood xylan as sole carbon source for wild-type, Δ*VmXyl1* and *C*Δ*VmXyl1* strains after 3 days of growth. The xylanase activity was expressed as units per min per ml (U/ml). **(B)** The xylanase activity of wild-type, Δ*VmXyl1* and *C*Δ*VmXyl1* strains recorded at different time intervals during infection. The xylanase activity was expressed as units per min per gram of fresh weight (U/g⋅FW).

We determined endo-β-1,4-xylanase activity in the culture filtrates of wild-type and mutant strains. Both beechwood xylan and apple branch extract induced the synthesis and secretion of xylanase. The gene deletion strain Δ*VmXyl1* exhibited 50 and 53% reduction in the xylanase activity in apple branch extract and beechwood xylan media, respectively. The retransformation with the native gene restored xylanase activity to wild-type levels (**Figure 7A**).

To test whether the lack of VmXyl1 affects the xylanase activity during fungal infection, we inoculated apple twigs with wild-type and mutant strains. We examined xylanase activity from 0 to 14 dpi. The deletion of *VmXyl1* caused a significant decrease in the xylanase activity (**Figure 7B**). The mutant Δ*VmXyl1* exhibited 44–83% reduction in the enzyme activity than wild-type at different time intervals; whereas the retransformation with the native gene restored the phenotype. These results are consistent with the effect of *VmXyl1* deletion mutant on xylan utilization. In order to understand the function of other endoxylanase genes in *V. mali*, the genome of *V. mali* were partially sequenced by the BGI Tech (Shenzhen, China). Preliminarily genomic data analysis revealed seven other putative endo-xylanase genes which have complete gene sequences (data unpublished). Thus, additional xylanase genes are likely to compensate for the loss of *VmXyl1* function in *V. mali*. Interestingly, the expression of the other seven xylanase genes of *V. mali* was not upregulated upon the *VmXyl1* deletion (Supplementary Figure S3).

## Discussion

Endoxylanases are among the main CWDEs that are secreted by pathogenic fungi, and thus play a key role in pathogen invasion, establishment, and replication in the host plants (Brito et al., 2006; Nguyen et al., 2011; Yu et al., 2016). The apple tree canker pathogen *V. mali* infects host plants through wounds, and then penetrates extensively into phloem and xylem tissues. However, the role of endoxylanases in pathogen virulence is still unknown; even the xylanase-encoding genes are not identified at present. In this study, we identified a novel endoxylanase gene *VmXyl1* from *V. mali* that contained a GH10 glycosyl hydrolase motif with xylanase activities. To our knowledge, we are the first to describe the endoxylanase gene in *V. mali* and its role during pathogen infection.

The putative mature protein VmXyl1 shares the characteristics of endoxylanase family GH10 that mainly include low pI, high molecular weight, and multiple conserved motifs (Collins et al., 2005; Chen et al., 2014). The proteins from this family are reported in some fungal pathogens such as *B. cinerea*, *Magnaporthe oryzae*, *Fusarium graminearum*, and *F. oxysporum* (Gómez-Gómez et al., 2001; Nguyen et al., 2011; Sella et al., 2013; García et al., 2017). The secretion of lytic enzymes is considered to be one of the main mechanisms by which *V. mali* destroys and overcomes the primary physical barriers against invading pathogen (Chen et al., 2012). Some enzymes such as pectate lyases and polygalacturonases can facilitate the invasion and colonization of host tissue by *V. mali* (Xu et al., 2016; Xu C.J. et al., 2017). Here, we studied the xylanase activity of VmXyl1, and demonstrate the role of endoxylanases in the virulence of *V. mali* on apple tree.

Interestingly, the expression pattern of VmXyl1 showed similarity to the gene that regulates the conversion of cell wall into low-molecular-weight easily assimilable sugars. Also, it was induced by the availability of beechwood xylan and not by the glucose. Previously, few studies have shown the expression of fungal xylanases using xylan, such as *xyn11A* of *B. cinerea*, and *xyl5* of *F. oxysporum*, whereas *xyl5* was detected only on oat spelt xylan but not on larchwood xylan (Gómez-Gómez et al., 2001; Brito et al., 2006). Here, beechwood, but not oat spelt xylan, induced the *VmXyl1*. The results indicate a considerable degree of specificity in the substrate regulation of *VmXyl1* expression, which may be due to the structural diversity of plant xylans (Gómez-Gómez et al., 2001).

The *VmXyl1* also showed a high level of expression during the infection of *V. mali*, which supports a role for this gene in pathogenicity toward apple. The *VmXyl1* expression pattern *in planta* was not significantly different between LXS080601 and other virulent strains of *V. mali* (data not shown). The *VmXyl1* expressed at the very early stage of fungus–plant interaction whereas its level of expression increased with lesion grade at 72–168 hpi. High expression of *VmXyl1* in the detached twigs of apple tree was in line with the xylanases activity during the infection of *V. mali*.

Although much effort is devoted to elucidating the biological roles of endoxylanases in pathogenic fungi by gene disruption (Apel et al., 1993; Gómez-Gómez et al., 2001; Wu et al., 2006), most of those studies failed to show the role of endoxylanases in the virulence of phytopathogens such as fungi. Interestingly, the deletion of *VmXyl1* caused more than 60% reduction in the lesion size, thus, confirming the role of this gene in the virulence of *V. mali*. In *B. cinerea*, *xyn11A* encodes an endo-β-1,4-xylanase Xyn11A, while disruption of this gene had a more pronounced effect on the virulence (Brito et al., 2006). It is perceived that Xyn11A determines fungal virulence via its necrotizing rather than catalytic activity (Noda et al., 2010). *In S. sclerotiorum*, a deletion mutant of endoxylanase SsXyl1 lost its virulence toward host plants; however, whether SsXyl1 has necrotizing activity is still unknown (Yu et al., 2016). The two endoxylanases Xyn11A and SsXyl1 belong to the family GH11, while the biological roles of endoxylanases from family GH10 in the virulence are rarely studied. Therefore, whether VmXyl1 contributes to the virulence via necrotizing activity needs to be investigated further.

In previous studies, the deletion of *SsXyl1* in *S. sclerotiorum* significantly altered its vegetative growth (Yu et al., 2016). In our case, *VmXyl1* showed a slight effect on mycelia density and the apical extension; however, the mycelial growth rate and mycelium dry weight did not differ statistically between the gene deletion mutant and the wild-type strain. Interestingly, our results revealed a role of *VmXyl1* in the formation of pycnidia. Since conidia production from pycnidia is a key phase in the fungal life cycle, the *VmXyl1* deletion mutants showed a 37% reduction in the number of pycnidia. Recently, Pérez-Hernández et al. (2017) identified a β-glucosidase gene *Bcsun1* from *B. cinerea*, and reported that the gene deletion affected in the production of conidia and sclerotia. In another study, Wu et al. (2017) investigated a mitogen-activated protein kinase gene *VmPmk1* in *V. mali*. The *VmPmk1* contributed to the fungal virulence by regulating CWDEs expression whereas its disruption impaired the production of pycnidia in the mutant strains. Thus, to the best of our knowledge, this is the first report to show that the endoxylanase is involved in conidia production.

In phytopathogenic fungi, redundancy of CWDEs genes in the genome represents a great challenge to illustrate the function of candidate genes via disruption (Walton, 1994; Xu M. et al., 2017). For instance, in *F. oxysporum*, endoxylanase genes such as *xyl3*, *xyl4*, and *xyl5* were disrupted and the resulting strains still retained full virulence (Gómez-Gómez et al., 2002). In *F. graminearum*, the deletion of the transcription factor *Xyr1* reduced endoxylanase genes expression and xylanase activity but did not influence fungal virulence (Sella et al., 2016). Similarly, in *M. oryzae*, mutation of even 10 endoxylanase genes did not abolish the virulence of mutant strains (Nguyen et al., 2011). In another study, the deletion of polygalacturonase genes (*Vmpg7* and *Vmpg8*) and pectate lyase gene (*Vmpl4*) had a weak effect on virulence, probably due to the expression or upregulation of other genes from the same family in the mutants (Xu et al., 2016; Xu C.J. et al., 2017). Interestingly, our data showed that the expression of other seven endoxylanase genes from the family GH10 and GH11 was not upregulated upon *VmXyl1*deletion (Supplementary Figure S3). No induction of other xylanase genes upon *VmXyl1*deletion indicated that these genes were not likely to complement the function of the deleted gene. The virulence of *VmXyl1* deletion mutants was reduced up to 60% as compared to the wild-type strain. It is thus very likely that VmXyl1, in addition to its enzymatic activity, may induce necrosis in host cells and thus contributes pathogenesis (Ron and Avni, 2004; Noda et al., 2010).

Moreover, a 50% reduction in the xylan utilization and endoxylanase production is consistent with a reduced virulence of the *VmXyl1* deletion mutants. Similarly, in *B. cinerea*, the deletion of *xyn11A* caused a 30 and 70% reduction in the xylanase activity and virulence, respectively (Brito et al., 2006). In contrast, deletion of triple genes *XYL1*, *XYL2*, and *XYL3* from *Cochliobolus carbonum*, demonstrated a 90% reduction in its endoxylanase activity; but the strain did not lose virulence toward host plants (Apel-Birkhold and Walton, 1996). Taken together, these data indicate the contribution of endoxylanase enzymatic activity to fungal virulence may depend on pathogen types.

In summary, we demonstrate that *VmXyl1* showed no effect on the colony morphology and mycelia growth whereas it significantly influenced pycnidia formation, xylan utilization and virulence toward apple tree. Further investigation of virulence factors in *V. mali* could yield valuable information for developing plant protection strategies.

## Author Contributions

BL and CW: conceived and designed the experiments. CY, TL, and XS: performed the experiments and analyzed the experimental data. CY, MS, and WL: contributed reagents/materials/analysis tools. CY, TL, and MS: wrote the paper. All authors participated in the editing and approved its final version.

## Conflict of Interest Statement

The authors declare that the research was conducted in the absence of any commercial or financial relationships that could be construed as a potential conflict of interest.
