# Supplementary material for: Deletion of Endo-β-1,4-Xylanase VmXyl1 Impacts the Virulence of Valsa mali in Apple Tree
Source: Front Plant Sci. 2018 May 17;9:663. doi: 10.3389/fpls.2018.00663 (PMC5966579; doi:10.3389/fpls.2018.00663)
Supplement: Supplementary file 1 [file Table_1.DOCX]

**Supplementary Table**

**Supplementary Table S1. Primers used in this study**

| **Fragments Use** | **Primers** | **Sequence (5'-3')** |  |
| --- | --- | --- | --- |
| Open reading frame | *VmXyl1*F | ATGCACTTCTCTTCTCACGC | |
|  | *VmXyl1*R | TTAAGCAGACATGGTCACGT | |
| 3'-end cDNA | Outer Primer | TACCGTCGTTCCACTAGTGATTT | |
|  | Inner Primer | CGCGGATCCTCCACTAGTGATTTCACTATAGG | |
|  | OT3RC | AGTCATCTTCTACTTCCTCCG | |
|  | IN3RC | CAAGACATGCGCAGTGAAGAAGACCAAAACT | |
| 5'-end cDNA | UPM | CTAATACGACTCACTATAGGGCAAGCAGTGGTATCAACGCAGAGT | |
|  | NUP | AAGCAGTGGTATCAACGCAGAGT | |
|  | GSP1 | TGTCATCGAGCTCGCCGTTGTCTGTC | |
|  | NGSP1 | CAGCTGGTTGAGTTGGGCATGG | |
| qRT-PCR | qRT-*VmXyl1*F | GCCGTCCTCAGCGACTCCA | |
|  | qRT-*VmXyl1*R | CGGTATAGCTGAACGTCCCAT | |
|  | *EF1-α*F | TGAGTTCGAGGCTGGTATCTCCAA | |
|  | *EF1-α*R | TGTCCATCTTGTTGATGGCGACGA | |
|  | *918F* | TCTCGATACGCTCAGCAACTGG | |
|  | *918R* | TCGCTGTTCGTGACATCCTCC | |
|  | *1425F* | CCTAGTAACGCAGCCGACCA | |
|  | *1425R* | ACCAATAATCTTGCCGTTCCAG | |
|  | *1896F* | TCGACAAAGCCTCAACGGAT | |
|  | *1896R* | AGTCAGACTCGTCATCGTCCA | |
|  | *1050F* | AGTAAGTTCTCTTGCACCCTT | |
|  | *1050R* | AGACGACGTAAAAGTCAGCTTC | |
|  | *1296F* | CTTTCTGACTCCGCCTACGTT | |
|  | *1296F* | AATTCGGTGGCATTTGACCT | |
|  | *1287F* | CGCCTCTTCGCACAACAACCACT | |
|  | *1287R* | ACCCCAGCTTCCGTATCGTCT | |
|  | *681F* | GGCACCCAGACGTTTAACCAG | |
|  | *681R* | GCTGCTCTCATAGCCCTCGGTA | |
| Deletion of *VmXyl1* | *VmXyl1*PF | GTCATCCTGATTCCGTGGCTGACTT | |
|  | *VmXyl1*PR | *TCTTGTCAAACGACACAAATTTTGTG*GGTGAAGAGCTTGCTTCAACTAT | |
|  | *VmXyl1*TF | *GAAGTACTCGCCGATAGTGGAAAC*GTTAGCAAGCCCTTTTTTAAA | |
|  | *VmXyl1*TR | CTCTGCGAGCGGTGACAGCTTGATC | |
|  | *HPH*F | CACAAAATTTGTGTCGTTTGACAAGATGG | |
|  | *HPH*R | GTTTCCACTATCGGCGAGTACTTCTACACAG | |
| Complementation of *VmXyl1* | *VmXyl1*CF | CCGGAATTCGTCATCCTGATTCCGTGGCT | |
|  | *VmXyl1*CR | CCGCTCGAGGGTGAAGAGCTTGCTTCAAC | |
