# Supplementary material for: Deletion of Endo-β-1,4-Xylanase VmXyl1 Impacts the Virulence of Valsa mali in Apple Tree
Source: Front Plant Sci. 2018 May 17;9:663. doi: 10.3389/fpls.2018.00663 (PMC5966579; doi:10.3389/fpls.2018.00663)
Supplement: Supplementary file 2 [file Image_1.pdf]

Supplementary material

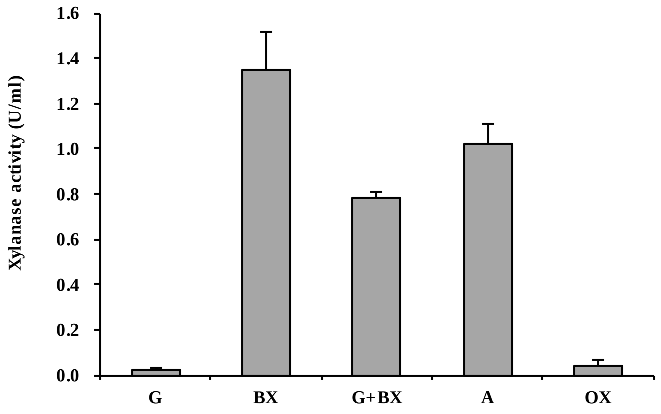

FIGURE S1 | The xylanase activity produced by wild-type LXS080601 in medium containing different carbon sources after 3 days of growth. The bars (G, BX, G+X, A, and OX) represent glucose, beechwood xylan, glucose+beechwood xylan, apple branch extract, and oat spelt xylan, respectively. The xylanase activity was expressed as units per min per ml (U/ml).

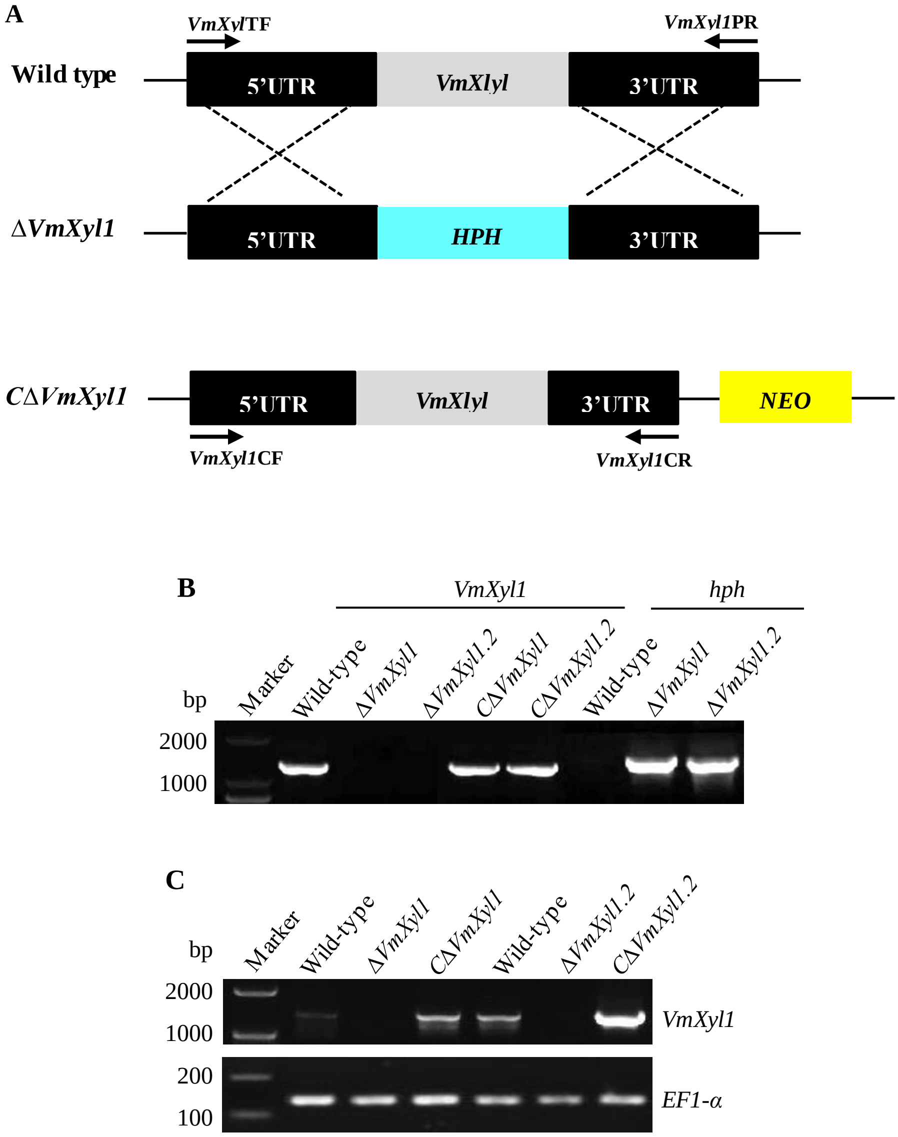

FIGURE S2 | Generation of *VmXyl1* deletion and complementation mutants. (A) Schematic diagram of gene disruption and complemented constructs. **(B)** Mutants were confirmed by genomic PCR with the primer pair *VmXyl1*F/*VmXyl1*R. **(C)** The RT-PCR analysis of *VmXyl1* expression with the primer pair *VmXyl1*F/*VmXyl1*R in wild-type, gene deletion (Δ*VmXyl1* and Δ*VmXyl1.2*), and complemented (CΔ*VmXyl1* and CΔ*VmXyl1.2*) strains. The expression of *EF1-α* was the internal control with the primer pair *EF1-α*F/*EF1-α*R.

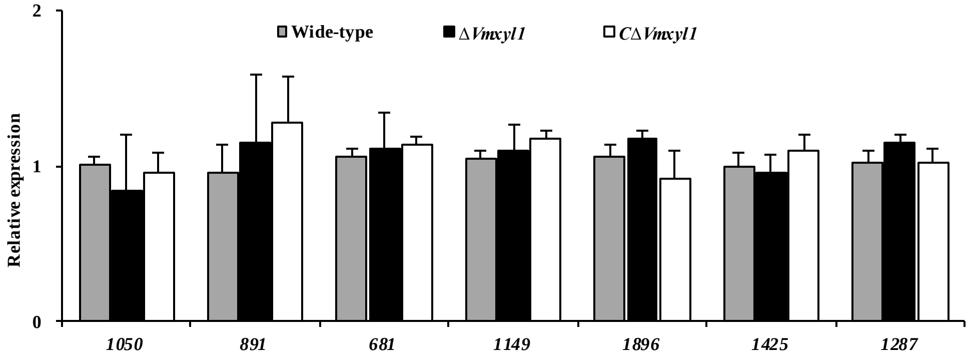

FIGURE S3 | The expression of endoxylanase genes in wild-type, *VmXyl1* deletion and complemented stains. Relative expression of seven endoxylanase genes from family GH10 and GH11 in the lesion border of apple tree bark were compared between wild-type and *VmXyl1* mutant strains at 5 dpi. The expression of *EF1-α* was used to normalize in each sample. Transcript levels of wild-type strain were normalized to one. The means and standard deviation of the transcription levels were calculated of three independent biological replicates. Bars represent the standard error.
